# Study of aqueous humour inflammatory mediators’ levels in a cohort of Egyptian patients with diabetic macular oedema

**DOI:** 10.1186/s12886-023-03192-w

**Published:** 2023-11-14

**Authors:** Amir Ramadan Gomaa, Ahmed Magdy Bedda, Hesham Fouad ElGoweini, Raghda Saad Zaghloul Taleb, Ahmed Mahmoud Abdelrahman Saleh

**Affiliations:** 1 Ophthalmology Department, Faculty of Medicine, Alexandria University, Alexandria, 21517 Egypt; 2 Clinical and Chemical Pathology Department, Faculty of Medicine, Alexandria University, Alexandria, 21517 Egypt

**Keywords:** Aqueous humour, Diabetes Mellitus, Inflammation, Macular Oedema

## Abstract

**Background:**

The aim was to study aqueous humour inflammatory mediators’ levels in a cohort of Egyptian patients with diabetic macular oedema (DMO).

**Methods:**

This was a case-control prospective study conducted on 2 groups: 25 eyes of 22 (11 females) patients seeking treatment for DMO as patients group, and 10 eyes of 10 (4 females) cataract patients as a control group. Aqueous humour was aspirated before intravitreal injection (patients’ group) or cataract surgery (control group). Inflammatory mediators in aqueous humour were measured using a multiplex bead immunoassay kit of 27 pre-mixed cytokines.

**Results:**

Eotaxin, interferon gamma-induced protein 10 (IP-10), monocyte chemoattractant protein-1 (MCP-1/CCL2) and interleukin-8 (IL-8/CXCL8) were found significantly higher in patients’ group compared to control group (*p* = 0.043, 0.037, 0.001, 0.015 respectively). On the contrary, interferon-gamma (IFN-gamma) and granulocyte colony-stimulating factor (G-CSF) were found significantly higher in control group than patients’ group (*p* = 0.003, 0.019 respectively). Basic fibroblast growth factor (Basic-FGF/FGF-2) and interleukin-1 receptor antagonist (IL-1ra) were found higher (but not statistically significant) in controls (*p* = 0.100 and 0.070 respectively). Additionally, a negative and significant correlation was found between Eotaxin level in aqueous humour and central macular thickness.

**Conclusions:**

Some mediators might be implicated in the pathogenesis of DMO either augmenting or suppressing role. Eotaxin, IP-10, MCP-1 and IL-8 might have a role in cases not responding to standard anti-vascular endothelial growth factor (VEGF) therapy. IL-1ra might have a protective role; therefore, the effectiveness of intravitreal injection of IL-1ra homologue needs to be studied in future clinical trials.

## Background

Diabetic macular oedema (DMO) is a well-known sight-threatening complication of diabetes mellitus (DM) [1]. Risk factors of diabetic retinopathy (DR) and DMO may include: increased HbA1c, hypertension, dyslipidemia, cataract surgery, pregnancy and duration of DM. Additionally, type 2 DM, sleep apnea, nephropathy and African-American ethnicity might be risk factors for DMO, while myopia might be a protective factor [2]. Pathophysiology of DR and DMO has been investigated over decades and concluded that vascular endothelial growth factor (VEGF) plays an important role [3]. Consequently, anti-VEGF treatments were developed to halt its negative effect on the retina and to improve vision [4]. Yet, not all cases responded well to anti-VEGF treatments [5, 6]. Further investigations continued to investigate other mediators released during DR/DMO trying to explore any significant contribution to the pathophysiology of DMO [7].

Several theories were proposed for DR pathophysiology. These could be categorized under two large entities: structural/cellular changes and molecular changes [8]. The most prominent structural and cellular changes are neurovascular unit affection, outer blood retinal barrier affection, diabetic cell death (apoptosis, pyroptosis and autophagy) [9] and finally, inflammation and altered repair mechanism [10]. The release of inflammatory mediators, beside the structural and cellular changes is thought to be mediated by intracellular molecular pathways [8]. Some major pathways were investigated extensively and proved to be implicated in the pathophysiology of DR to a great extent, which are: polyol pathway, advanced glycation end-products formation, protein kinase-c pathway, and finally hexosamine pathway. Those pathways are believed to be connected by a unifying mechanism [11]. Other pathways and factors were also investigated such as: hypoxia-inducible factor 1α (HIF1α) [8], angiopoietins (Ang2-Tie2 pathway), renin-angiotensin-aldosterone system [10], kallikrein-kinin system (KKS) [8], sirtuins, and high-mobility group box-1 (HMGB1) protein. Oxidative stress and redox reactions are thought to be part of this unified mechanism [12].

The released inflammatory mediators serve as crosstalk between cells (immunological or non-immunological). DR and DMO are thought to represent a low-grade inflammatory state, thus several inflammatory mediators were explored trying to find their role in pathophysiology of DR [10, 13]. Pro-inflammatory mediators are mainly grouped into angiogenic or growth factors, chemokines and cytokines. VEGF is a well-known angiogenic factor which plays a significant role in DR and DMO. Examples of those mediators (or biomarkers) that related to ocular tissue are: pro/anti-inflammatory interleukins such as interleukin-1 receptor antagonist (IL-1ra), IL-1B, IL-6 and IL-8, granulocyte macrophage colony-stimulating factor (GM-CSF), tumor necrosis factor-alpha (TNFα) and interferon-gamma (IFN-gamma). Chemokines like Eotaxin-1, monocyte chemotactic protein-1 (MCP-1), macrophage inflammatory protein-1 alpha (MIP-1α), macrophage inflammatory protein-1 beta (MIP-1β) and regulated on activation, normal T-cell expressed and secreted (RANTES). Angiogenic and growth factors like VEGF, placental growth factor (PIGF), basic-fibroblast growth factor (basic-FGF) and platelet derived growth factor-β (PDGF- β) [14].

In the present study, we measured aqueous humour levels of inflammatory mediators in a cohort of Egyptian patients with DMO and also investigated the correlation between cytokines and central macular thickness. To our knowledge, this is the first study to investigate the aqueous levels of 27 cytokines in Egyptian patients with DMO.

## Methods

### Study participants

A prospective case-control study was conducted on 25 eyes of 22 patients seeking treatment for DMO at Ophthalmology Department, Alexandria University Main Hospital, Alexandria, Egypt between March and August 2022. 10 eyes of 10 age and sex matched healthy subjects without ocular diseases other than cataract were included as a control group. Sample size was calculated using G*Power 3.1.9.2 software (Heinrich Heine University Düsseldorf, Germany) based on previous studies at a power of 80%, 0.05 α error and allocation ratio 2.5. Full ocular, medical and family history was taken. Full ophthalmic clinical examination was performed for all groups including: slit lamp examination, dilated fundus examination, applanation intraocular pressure measurements, cycloplegic refraction and uncorrected and best corrected visual acuity (BCVA). For Patients with DMO, ocular coherence tomography (OCT) and fundus fluorescein angiography (FFA) were performed for assessment of DMO and DR. Both groups did not undergo ocular surgery before nor had any other ocular disease or systemic disease that could induce ocular inflammatory state.

The inclusion criteria for the study group were as follows: (1) age ≥ 40 years; (2) DMO with OCT central thickness > 300 μm; (3) BCVA 3/60 or better; (4) non-proliferative diabetic retinopathy (NPDR) (mild-moderate). Exclusion criteria were as follows: (1) previous treatment for DMO (laser or anti-VEGF); (2) macular degeneration; (3) macular epiretinal membrane, or vitreomacular traction, (4) macular oedema due to other causes; (3) concomitant glaucoma or ocular hypertension; (4) previous eye surgery; (5) uveitis; (6) any other ocular or systemic disease that is thought to affect mediators’ level.

### Aqueous humour sampling

Aqueous humour (about 0.2 ml) was aspirated before intravitreal injection (patients group) or cataract surgery (control group), through limbal incision using 30-gauge needle. Small air bubble was injected before aspiration to keep anterior chamber formed after aspiration. The samples were immediately frozen and stored at -80 °C until analysis.

### Inflammatory mediators measurement

A kit of 27 pre-mixed cytokines was used for analysis (Bio-Plex Pro Human Cytokine 27-plex assay; #M500KCAF0Y, Bio-Rad, Hercules, CA, USA). The samples were diluted 1:4 using Bio-Plex sample diluent. The measured cytokines were: IL-1ra, IL-1b, IL-2, IL-4, IL-5, IL-6, IL-7, IL-8, IL-9, IL-10, IL-12, IL-13, IL-15, IL-17, IFN-gamma, basic-FGF, granulocyte colony-stimulating factor (G-CSF), GM-CSF, interferon inducible protein-10 (IP-10), MCP-1, MIP-1α, MIP-1β, PDGF-β, CC chemokine ligand-3 (Eotaxin), RANTES, TNFα, and VEGF. The assay procedure was performed according to the manufacturer’s instructions. Aqueous humour samples, standards and control were assayed in duplicate. Standard curves for cytokines were generated using Bio-Plex™ Manager Software (version 6.2). Mediators’ concentrations in samples were determined using a 5-parameter logistic curve. Final concentrations were calculated from the mean fluorescence intensity and expressed in pg/ml.

### Statistical analysis

Data were analyzed using IBM® SPSS® Statistics version 20.0. (SPSS Inc, IBM, Chicago, Illinois, USA). Categorical data were described as numbers and percentages. Fisher’s exact test was performed to determine association between two categorical variables. Kolmogorov-Smirnov test was performed to verify the normality of distribution for each variable. Numerical data were described using range (minimum and maximum), mean, and standard deviation for normally distributed variables or range (minimum and maximum), median and interquartile range (IQR) for abnormally distributed ones. Student’s t-test was performed to compare between two normally distributed numerical variables. While on the contrary, for abnormally distributed numerical variables, Mann-Whitney U test was performed to compare between the two studied groups. Pearson coefficient was used to correlate between aqueous levels of inflammatory mediators and central macular thickness. To assess the correlation between inflammatory mediators, Spearman’s rank-order correlation coefficients were calculated. The BCVA values were converted to logarithm of the minimum angle of resolution (logMAR).The accepted level of significance was stated at 0.05 (*P* ≤ 0.05 was considered significant).

## Results

### Demographic and clinical data

The study included 35 eyes, 25 eyes of 22 patients (11 males and 11 females) with DMO and 10 eyes of 10 cataract patients (6 males and 4 females) as a control group. The demographic and clinical characteristics of the study groups are summarized in Table 1.

**Table 1:** Demographic and clinical characteristics of the study groups (*n* = 32 participants/35 eyes)

	Patients (*n* = 22 patients/25 eyes)	Control (*n* = 10)	*p*-value
**Gender, *n (%)***			
Male	11 (50)	6 (60)	0.712^a^
Female	11 (50)	4 (40)	
**Age (years)**			
Range	40.0–71.0	48.0–73.0	0.860^b^
Mean ± SD	60.50 ± 9.35	59.90 ± 7.65	
**Type of DM**, ***n (%)***			
Insulin dependent	5 (22.7)	N/A	
Non insulin dependent	17 (77.3)	N/A	
**Duration of DM (years)**			
Range	4.0–20.0	N/A	
Mean ± SD	12.50 ± 5.75	N/A	
**BCVA (LogMAR)**			
Range	0.10–1.30	0.20–1.30	0.895^b^
Mean ± SD	0.68 ± 0.32	0.70 ± 0.32	
**IOP**, ***mm Hg***			
Range	10.0–22.0	11.0–21.0	0.854^b^
Mean ± SD	16.56 ± 3.37	16.80 ± 3.71	
**FFA findings**			
** Leakage pattern**, ***n (%)***			
Diffuse	11 (44%)	N/A	
Focal	14 (56%)	N/A	
** Macular ischaemia**, ***n (%)***			
Absent	21 (84%)	N/A	
Present	4 (16%)	N/A	
**OCT findings**			
** Central macular thickness**, ***µm***			
Range	309.0–663.0	N/A	
Mean ± SD	434.0 ± 88.92	N/A	
** Macular cysts**, ***n (%)***			
Spongiform	5 (20)	N/A	
Small	5 (20)	N/A	
Moderate	7 (28)	N/A	
Schisis	8 (32)	N/A	
** Subretinal fluid**, ***n (%)***	8 (32)	N/A	
** DRIL**, ***n (%)***	9 (36)	N/A	
** EZ/ELM**, ***n (%)***			
Intact	17 (68)	N/A	
Disrupted	8 (32)	N/A	

^a^Fisher’s exact test

^b^Student’s t-test

*BCVA *Best corrected visual acuity, *DM *Diabetes mellitus, *DRIL *Disorganization of retinal inner layers, *EZ/ELM *Ellipsoid zone/extend limiting membrane, *FFA *Fundus fluorescein angiography, *IOP *Intraocular pressure, *OCT *Ocular coherence tomography

### Inflammatory mediators measurements in aqueous humours

Assayed inflammatory mediators with a detection rate of 70% or more in total number of samples were chosen for statistical analysis. Eight mediators passed this detection rate: Eotaxin, IP-10, MCP-1, IFN-gamma, Basic-FGF, IL-8, G-CSF and IL-1ra. Eotaxin, IP-10, MCP-1 and IL-8 were found significantly higher in DMO patients compared to the control group (*p*-value = 0.043, 0.037, 0.001 and 0.015 respectively). IFN-Gamma and G-CSF were found significantly lower in DMO patients compared to the control group (*p*-value = 0.003 and 0.019 respectively). Basic-FGF and IL-1ra were insignificant between both groups (*p*-value = 0.1 and 0.07 respectively). Table 2 illustrates the concentrations of included inflammatory mediators.

**Table 2:** The concentrations of inflammatory mediators in aqueous humours of study groups (pg/ml)

	Patients (*n* = 22 patients/25 eyes)	Control (*n* = 10)	*p*-value^a^
**Eotaxin**			
Range	2.59–16.22	2.02–5.78	0.043^*^
Median (IQR)	5.27 (4.11–7.50)	4.32 (2.02–5.16)	
**IP-10**			
Range	0.0–3530.16	52.02–509.38	0.037^*^
Median (IQR)	213.02 (151.75–335.65)	136.35 (94.31–169.1)	
**MCP-1**			
Range	121.89–1205.45	89.81–446.45	0.001^*^
Median (IQR)	213.63 (162.54–292.0)	130.46 (96.38 - 138.97)	
**IFN-gamma**			
Range	0.0–6.21	0.78–3.22	0.003^*^
Median (IQR)	0.52 (0.0–1.13)	1.51 (1.32–2.80)	
**Basic-FGF**			
Range	0.0–56.65	0.0–40.08	0.100
Median (IQR)	18.24 (0.0–30.26)	30.26 (30.26–40.08)	
**IL-8**			
Range	0.0–47.86	0.0–35.52	0.015^*^
Median (IQR)	16.96 (6.94–31.27)	3.45 (0.0–6.94)	
**G-CSF**			
Range	0.0–46.03	9.04–65.37	0.019^*^
Median (IQR)	4.23 (0.0–14.68)	13.57 (11.33–22.21)	
**IL-1ra**			
Range	0.0–1351.34	93.27–382.12	0.070
Median (IQR)	149.65 (0.0–298.27)	266.64 (193.9–327.79)	

^a^Mann-Whitney U test

*Statistically significant at *p* ≤ 0.05

### Correlation between inflammatory mediators concentrations and central macular thickness

**Fig. 1:**
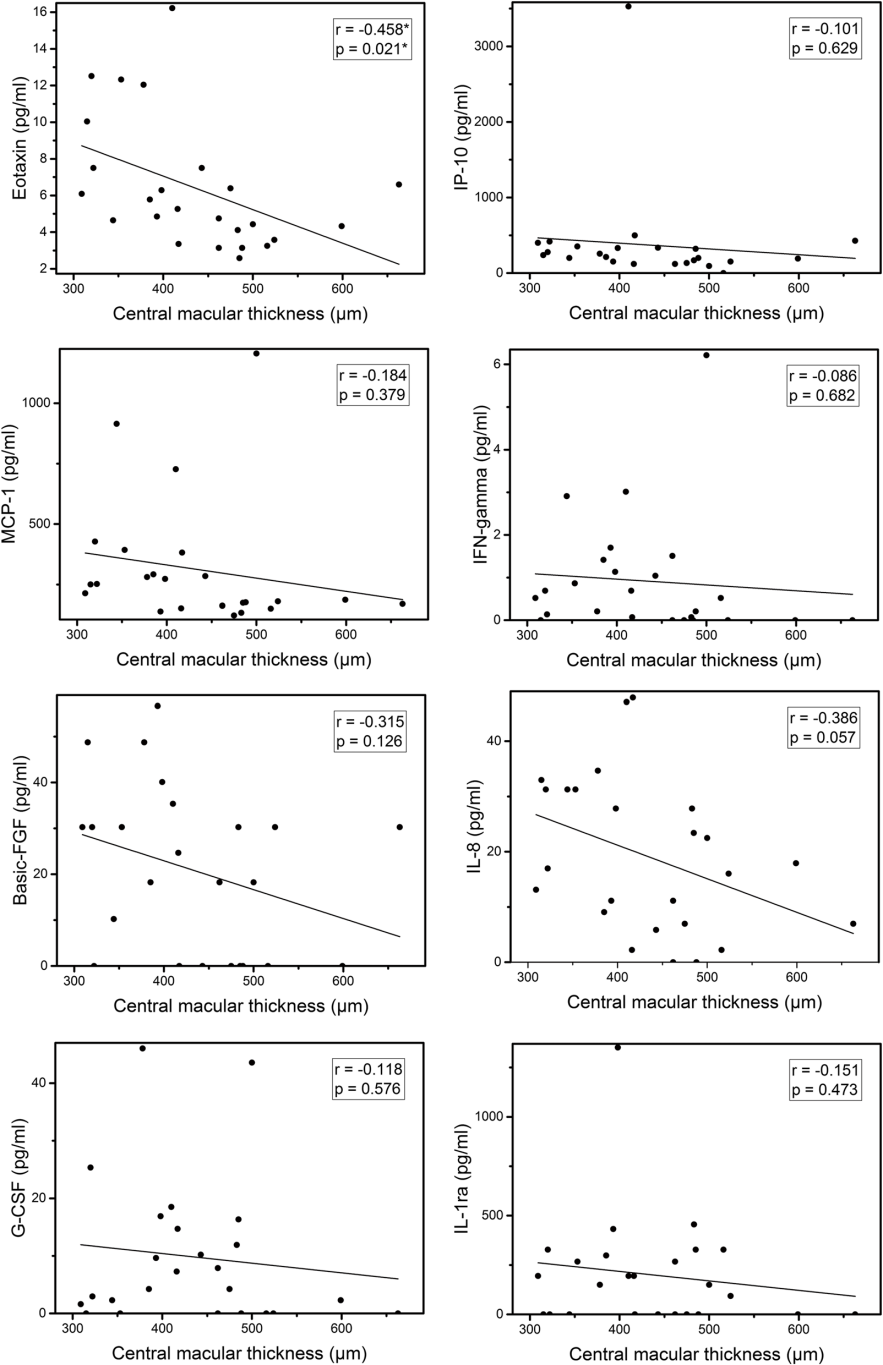
Scatter plots of the correlation between aqueous levels of inflammatory mediators and central macular thickness. Significant *p*-value is indicated by an asterisk. *r* = Pearson correlation coefficient

A negative and significant correlation was found between aqueous level of Eotaxin and central macular thickness (*p* = 0.021, *r*=-0.458). None of the other inflammatory mediators correlated significantly with central macular thickness. Figure 1 illustrates the correlation between different inflammatory mediators and central macular thickness.

### Correlation between the concentrations of inflammatory mediators

Table 3 shows that these inflammatory mediators are related, Eotaxin with IP-10 (rs = 0.505, *p* = 0.010), MCP-1 (rs = 0.405, *p* = 0.045) and basic-FGF (rs = 0.494, *p* = 0.012); IP-10 with MCP-1 (rs = 0.490, *p* = 0.013) and IL-8 (rs = 0.485, *p* = 0.014); MCP-1 with IFN-gamma (rs = 0.470, *p* = 0.018) and IL-8 (rs = 0.605, *p* = 0.001); IFN-gamma with G-CSF (rs = 0.429, *p* = 0.032) and IL-1ra (rs = 0.398, *p* = 0.049); basic-FGF with IL-1ra (rs = 0.414, *p* = 0.040); IL-8 with G-CSF (rs = 0.408, *p* = 0.043); G-CSF with IL-1ra (rs = 0.403, *p* = 0.046).

**Table 3:** Correlation between the concentrations of inflammatory mediators in DMO patients

		Eotaxin	IP-10	MCP-1	IFN-gamma	Basic-FGF	IL-8	G-CSF	IL-1ra
**Eotaxin**	**r**_**s**_		0.505^*^	0.405^*^	0.275	0.494^*^	0.322	0.226	0.011
	p		0.010^*^	0.045^*^	0.183	0.012^*^	0.116	0.278	0.958
**IP-10**	**r**_**s**_			0.490^*^	-0.031	0.138	0.485^*^	0.148	-0.130
	p			0.013^*^	0.885	0.510	0.014^*^	0.480	0.536
**MCP-1**	**r**_**s**_				0.470^*^	0.081	0.605^*^	0.333	-0.135
	p				0.018^*^	0.699	0.001^*^	0.104	0.521
**IFN-gamma**	**r**_**s**_					0.211	0.022	0.429^*^	0.398^*^
	p					0.310	0.918	0.032^*^	0.049^*^
**Basic-FGF**	**r**_**s**_						0.376	0.166	0.414^*^
	p						0.064	0.428	0.040^*^
**IL-8**	**r**_**s**_							0.408^*^	0.056
	p							0.043^*^	0.792
**G-CSF**	**r**_**s**_								0.403^*^
	p								0.046^*^
**IL-1ra**	**r**_**s**_								
	p								

r_s_: Spearman correlation coefficient

*Statistically significant at *p* ≤ 0.05

## Discussion

DMO is the most prevalent cause of moderate vision loss in diabetic patients. Multiple interacting cellular signaling cascades are involved in the pathogenesis of DMO. These cascades result in the retinal and microvascular abnormalities observed in DMO. Numerous cytokines were reported to be involved in the pathogenesis of DMO, and some of them might be useful as biomarkers to assess the severity of the disease [6]. The current study investigated levels of aqueous humour’s inflammatory mediators in DMO patients in a cohort of Egyptian population. Eotaxin (known also as Eotaxin-1 or chemokine 11 (CCL11)) [15] was significantly higher in patients with DMO than the control group. Several studies are consistent with our results, Wei et al. [16] and Srividya et al. [17] found Eotaxin significantly higher in DMO than controls in aqueous humour and vitreous humour respectively. Similarly, Ghodasra et al. [18] and Mastropasqua et al. [19] reported higher Eotaxin levels in vitreous humour and aqueous humour of DMO patients, respectively; however it failed to reach statistically significant level. Generally, Eotaxin level was high in diabetic patients either with or without DR/DMO than controls, but not significantly [20–22]. Importantly, Eotaxin also showed decreased levels significantly after treatment with anti-VEGF agents [23, 24], which may give a clue about its role in DMO.

IP-10 is a chemoattractant secreted by many cells like endothelial cells and macrophages; it shares in T-helper type 1 immune response [20]. In our study, we found significant increase of IP-10 in DMO than controls. Our results are supported by other researchers [16, 17, 25, 26]. MCP-1 is also a chemotactic mediator that attracts monocytes to secret growth factors like VEGF and affects microglia around vessels in DMO [27]. In our study, it was also significantly increased in DMO than controls. This finding is in agreement with previous studies [16, 25, 26, 28]. IL-8 is a chemokine that shares also in inflammation by attracting neutrophils and T-cells, even it is considered proangiogenic [16, 27]. It was found in our study that it is significantly high in DMO than controls similar to other researchers [17, 18, 25, 26, 28]. IL-1ra is a potent anti-inflammatory mediator and its therapeutic form (Anakinra) is used to treat systemic inflammatory conditions like arthritis [17, 29]. Its low level in DMO may be a clue for inflammatory process that couldn’t be buffered, which is proved in our study that shows lower levels in DMO than controls (but not significantly). Of note, Wei et al. [16] worked on naïve-DMO using the same multiplex kit provided by Bio-Rad laboratories and showed similar results. In contrast to our results, Srividya et al. [17] found statistically significant higher levels in DMO but they included non-naïve DMO. Others also found no significant differences and even very close levels in both groups [19].

In our study, IFN-gamma had significant lower levels in DMO than controls. In line with our results, Roh et al. [30] and Lee et al. [31] found also higher IFN-gamma level in controls but not significant, whereas other researchers did not agree with ours [17]. Nevertheless, others found no significant difference between DMO and controls [18]. Interestingly, many researchers failed to detect measurable levels of IFN-gamma in their samples [19, 20, 32], denoting the difficulty to detect its true level and consequently its role in DMO pathogenesis. Basic-FGF levels in our study were higher in controls but not significantly. Khuu et al. [33] showed similar results on naïve-DMO patients with non-proliferative DR, this was also supported by Ghodasra et al. [18], on contrary to Srividya el al [17] who found significant higher levels in non-naive DMO patients. Mastropasqua et al. [19] found significant higher levels in controls, which supports our results more. Moreover, Dong et al. [32] found higher levels in diabetics without DMO than those with it. Furthermore, Chen et al. [20] found lower levels in DR patients than diabetics without retinopathy. This raises the question if basic-FGF might have a protective role against DMO. In our study, G-CSF analysis revealed significant higher levels in controls than DMO. Khuu et al. [33] and Ghodasra et al. [18] found higher levels in controls but not significantly; whereas, Wei et al. [16] didn’t agree with this result and found significant higher levels in DMO patients. On the higher side of mediators’ levels in DMO than controls, Eotaxin, MCP-1, and IP-10 showed positive correlations with each other. The same finding was reported by Wei et al. [16] Consistent with previous studies [16, 32], IL-8 had positive correlation with MCP-1and IP-10, which may suggest their involvement in a common pathway. However, on the lower side, IL-1ra showed positive correlations with INF-gamma, G-CSF and basic-FGF, which may represent a suppressed protective pathway connecting them. IFN-gamma was also correlated positively with MCP-1 and G-CSF which in turn is positively correlated with IL-8. In addition, basic-FGF was positively correlated with Eotaxin.

Non-responders to regular anti-VEGF suffer from unnecessary repeated injections, increasing the risk for complications, financial burden, and finally, psychological depression. According to this study, cases of non-responders to regular anti-VEGF agents may have another unknown pathophysiological pathway contributing to DMO. This unknown pathway could be blocked by new agents similar to anti-VEGF agents.

Mediators’ levels were found to decrease with increasing central macular thickness in this study, although not significantly except for Eotaxin. This could be attributed to either small sample size or this could be related to the anti-inflammatory mechanisms triggered by retinal cells in response to excessive stretching of retinal layers by accumulated intraretinal fluid, trying to halt the continuous release of mediators to prevent further damage to the retina.

This study has some limitations, such as small sample size and absence of some clinical parameters such as hypertension, lipid profile and HbA1c levels due to lack or uncertain data provided by the patients, also poor compliance and financial status of patients. Cataract was also not included as a parameter in the study group due to different types and shapes that is evaluated subjectively; instead final visual acuity was taken as a relatively better objective reproducible way for correlation with mediators. However, this study is the first on Egyptian population. Besides, a point of novelty in our study was that all cases were naïve DMO on contrary to many previous studies that included mixed status of DMO.

## Conclusions

In conclusion, inflammatory process and mediators could be a part of DMO beside angiogenesis mediated by VEGF. This is supported by high levels of MCP-1, IL-8, IP-10 and Eotaxin, in addition to low level of protective mediators like IL-1ra. Further investigations are needed trying to find a pathway to be blocked in DMO not responding to VEGF, or even to work beside VEGF.

## Data Availability

Data are available upon reasonable request to the corresponding author.
